# Soil and vegetation conditions changes following the different sand dune restoration measures on the Zoige Plateau

**DOI:** 10.1371/journal.pone.0216975

**Published:** 2019-09-20

**Authors:** Jiufu Luo, Dongzhou Deng, Li Zhang, Xinwei Zhu, Dechao Chen, Jinxing Zhou

**Affiliations:** 1 Key Laboratory of State Forestry Administration on Soil and Water Conservation, Beijing Forestry University, Beijing, China; 2 Jianshui Research Field Station, Beijing Forestry University, Beijing, China; 3 Sichuan Academy of Forestry, Chengdu, China; 4 Sichuan Aba Forestry Science and Technology Research Institute, Wenchuan, China; Chinese Academy of Forestry, CHINA

## Abstract

Alpine sand dunes restoration is extremely difficult but important in the ecosystem restoration. Sand dunes are known as harsh soil and poor seed bank which freed from advantages on plants growth naturally. Effective restoration measures are required to guide the sand dune restoration. Here, indigenous grass (*Elymus nutans*) was sown in sand dune on the Zoige Plateau and treated with no sand barrier (CK) and environmental friendly materials including wicker sand barrier (wicker) and sandbag sand barrier (sandbag). The soil conditions were assessed by measuring the soil moisture and nutrients of the topsoil, and interspecific relationship and population niche were utilized to analyze the plant community structure variances among different restoration measures. Results showed that the soil and vegetation in the sand barriers measures were better than that in the CK. The soil moisture in the sandbag measure was 16.67% higher than that in the wicker measure. The nutrients content and microbial biomass were also the best in the sandbag measures. The ratio of strong association was the highest in the sandbag measure and the lowest in the CK, whereas the plants had the highest none association ratio in the CK. In addition, the average population niche overlap ranked by sandbag (0.39)>wicker (0.32)>CK (0.26). Thus, incorporation of sand barriers and indigenous grass seeding in alpine sand dunes could promote the sand dune restoration. And the sandbag measure showed a stronger improvement effect on the sand dune soil and vegetation conditions than the wicker measure.

## 1. Introduction

The terrestrial ecosystem has been experiencing increasing severe desertification [1]. Desertification threatens the ecological safety and its restoration is one of the vital elements in the mountain-river-forest-farmland-lake-grass system (referred as meta-ecosystem) restoration [2, 3]. The Zoige Plateau is part of the Qinghai-Tibet Plateau that claims to be the "Roof of the world" and "The Third Pole". The long-termed complex causes (e.g., overgrazing, climate change) accelerated sand dunes expanding process on the Zoige Plateau [4–6]. Sand dunes are known as covered by nutrient devoid sandy soil that freed from positive properties for plant growth [7]. The expanded sand dunes destroyed fertile land and had negative impacts on the livestock productivity, society development, ecological civilization, household income or human beings health [8–10]. Thus, it is badly in need to conduct sand dunes restoration in the alpine fragile zone.

Decades worth of works have been conducted on the sand dunes restoration [11, 12]. Mechanical sand barriers as the classical measure have been demonstrated to fix the sand dune well in the initial stages [13]. For example, stone checkerboard barriers and high sandbreak palisade have been utilized to fix the sand dunes along the Qinghai-Tibet railway [13, 14]. However, the single use of barriers is non-optimal choice in the alpine sand dune restoration because they are short using life, costly expense and hard construction. They were often buried by sand sediments due to vegetation cover lacking eventually [13].

Vegetation restoration is one of the biotic approaches and important objectives in the sand dune restoration [15, 16]. It has been proved to be practical in decreasing wind velocity and increasing soil nutrients in sand dune [17–19]. However, natural vegetation restoration is almost not feasible because of the poor seed bank [20, 21]. Hence, the indigenous seeds application is an inevitable method to improve seed bank [22, 23]. In Ille et Vilaine north Brittany coast of France, the marram grass (*Ammophila arenaria*) was one of an optimal species for sand dune restoration and the restoration was satisfactory in terms of the geomorphology [11]. Ambitious afforestation programme, such as ‘Tree-screens’ and ‘shelter-belt’ plantations, in the Thar Desert in India were launched which improved vegetation cover, reduced soil loss, and decreased wind velocity by 20–46% on the leeward side [17]. It was proved that *Hedysarum scoparium*, *Artemisia sphaerocephala* and *Artemisia wellbyi* have a good growth status on the sand dune in Tibet of China, indicating they could be used as pioneer plants in the vegetation restoration on the such sandy lands [24]. Masayuki et al. also found that the flourishing herbs could keep sand dune from reactivating in the semi-arid regions of Inner Mongolia where disagreed with crop growth [25]. In the Mu Us Sandy Land in China, farmland constructed has changed the barren desert to fertile farmland and vegetation cover increased from 24.5% to 74.3% through ten-year restoration. Moreover, shrub-planting (*Artemisia ordosica*) may develop to a typical steppe with the sand dune fixation process in this sandy land [26, 27].

These preliminary studies providing some evidence that abiotic or biotic measure could fix or restore the sand dune in a way. However, it remains uncertain whether they could work on the fragile alpine zone that dominated by windiness, harsh soil and droughty. Here, we focus on two environmental friendly barrier materials (i.e., Poly Lactic Acid sandbag and *Salix paraplesia* wicker) that are easily reproducible and durable in harsh conditions. Poly Lactic Acid is hydrophilic, ultraviolet radiation resistance and easy transportation [28], making them as optimal barrier materials in sand dune restoration on the Zoige Plateau. Meanwhile, *S*. *paraplesia* is widely cultivated in the alpine area which makes it convenient to acquire wicker materials. These two materials combined with indigenous grass (*Elymus nutans*) were used with expectation to fix the active alpine sand dune. Our objective is to compare the different alpine sand dune restoration measures effect and to provide a suitable strategy for sustainable sand dune restoration.

## 2. Materials and methods

### 2.1. Study area

The study area (102°55′E, 33°41′N; 3500 m above sea level) is located in Zoige County on the Zoige Plateau at the northeastern edge of the Qinghai-Tibet Plateau in China. The Zoige Plateau is also known as Zoige Basin, Zoige Wetland, Zoige Marshland and Zoige Grassland and covered with subalpine meadow and swamp meadow. It is characterized by an alpine continental monsoon climate and a pronounced winter season and short summer. The aeolian desertified lands area increased greatly on the Zoige Plateau, which accounting for 3.95% in 1975 and 13.09% in 2005. And the Zoige County is the most seriously influenced by the desertification which accounting for 65.70% of total desertification area of Zoige Plateau in 2005 [4]. The Zoige County is located at northeastern of the Zoige Plateau. The annual average temperature is about 0.7°C and the average annual rainfall ranges is 657 mm while the potential evaporation is more than 1200 mm. The mean January temperature is about -10.6°C and the mean July temperature is about 10.8°C [4]. The wind is northwest prevailing direction and the maximum wind speed is up to 36 m s^-1^ [9]. The soil is dominated by alpine or subalpine meadow soils, and peat moor soils. However, the aeolian sandy soil has caused serious threatens to rangeland or village of Zoige County.

This sand dune restoration study was conducted in the field of Zoige County in the Sichuan Province which is the State-owned Land. This land is the typical alpine sandy land on the Zoige Plateau. And it did not involve any rare, endangered and protected plants. Moreover, this sand dune restoration research was supported by the Department of Science and Technology, National Forestry and Grassland Administration. Thus, the government of Zoige County also gave us the permission to carry out this study in this land.

### 2.2. Field investigation design and sampling

**Fig 1:**
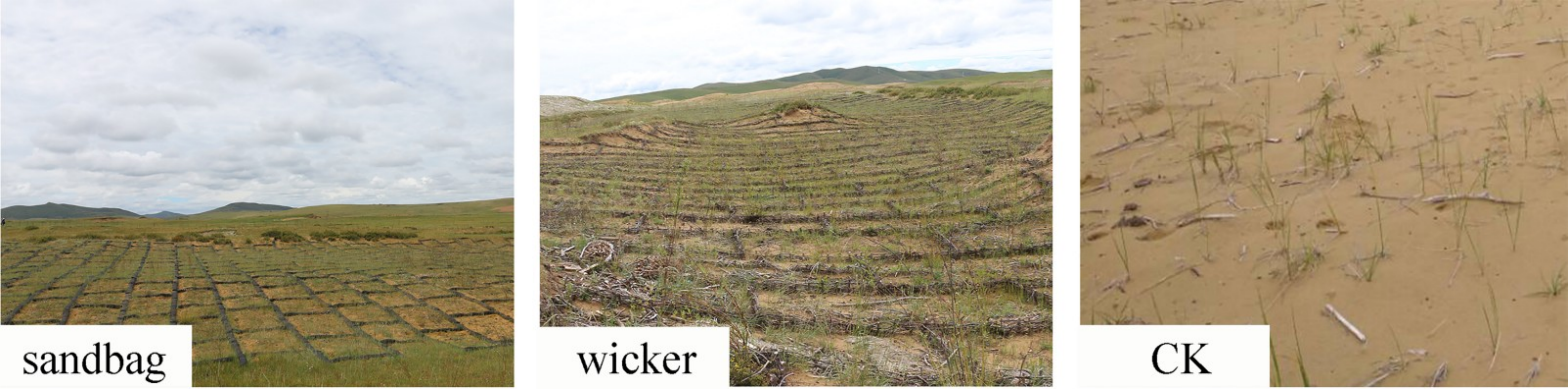
Demonstration area of different restoration measure.

We established a restoration demonstration zone in a 10 ha degraded land where the active sandy land occupied more than 55% in 2014. This area is one of the typical degraded alpine lands on the Zoige Plateau. The sand, silt and clay content of the sand dune soil were 96.10%, 0.10% and 3.80%, respectively. We sowed *Elymus nutans* (60 kg hm^-2^) in the sand dune that treated by sandbag sand barrier, wicker sand barrier and no sand barrier (referred as “sandbag”, “wicker” and “CK”) in April of 2014. All the sand barrier grid size was 2.0 m × 2.0 m, with the height of 0.3 m. The sandbags were filled up with the sand, which was collected from the local sand dune. The wicker sand barrier was woven by the 3 or 4-year-old willow trunk and 2 or 3-year-old wicker. The willow trunk was sawn into about 50 cm long sticks and inserted 20 cm of the sticks in the ground serving as anchor stakes. The primary belt of sand barrier was vertical with the main wind direction, and the secondary belt was vertical with the primary belt. Thus, the sandbag and wicker sand barriers were crisscross grids. Ploughing the land into about 5 cm depth for sowing and then compacting the land to protect the seed from being blown away. Three restoration measures consisting of sandbag, wicker, and CK were arranged in a randomized pattern with three replicates (about 0.4 ha per replicate) in the restoration demonstration zone (Fig 1).

12 quadrats (1.0 m ×1.0 m) were equally distributed in each replicate, and 3 replicates for each measure, so a total of 108 quadrats were investigated in all the three measures in August of 2016 (the third growth season). Parameters of plant taxa, height, coverage of each species and individual number were recorded (Table 1, S1 Dataset). Surface soil in each replicate was sampled by 12 soil cores, and sieved (<2 mm) them to filter out gravel or plant roots. Each of soil samples was divided into three subsamples. One subsample was saved in a refrigerator (4°C) for microbial biomass carbon (MBC) and microbial biomass nitrogen (MBN) determination by the chloroform (CHCl_3_) fumigation-incubation method co-applied with an N/C Analyser (multi N/C® 3100 TOC, analytikjena, Germany). One subsample was air-dried to measure total soil organic carbon (thereafter SOC) by K_2_Cr_2_O_7_-H_2_SO_4_ oxidation and titration using FeSO_4_. Total soil nitrogen (TN) was digested by H_2_O_2_-H_2_SO_4_ and total soil phosphorus (TP) was digested by HClO_4_-H_2_SO_4_ to get solutions. Soil ammonium nitrogen (AN) and soil nitric nitrogen (NN) were extracted by KCl and soil available phosphorus (AP) was extracted by NaHCO_3_ to get the solutions. These solutions were determined by Smartchem Discrete Auto Analyzer (Smartchem 200, AMS/Westco, Italy). In addition, the rest soil subsample was used for soil moisture determination using the gravimetrical method by drying at 105°C to achieve constant weight (S1 Dataset).

**Table 1:** Basic information of plots in the three measures in August of 2016.

	Number of individuals (m^-2^)	Vegetation coverage (%)	Average height of individuals (cm)	Community height (cm)	Main plants
**sandbag**	56.83±1.46c	19.08±0.77c	12.65±1.05a	39.97±0.47a	*Elymus nutans*
**wicker**	32.19±1.18b	11.58±0.70b	10.35±0.65a	28.58±0.47b	*Elymus nutans*
**CK**	21.25±1.25a	4.42±0.16a	8.52±0.49b	17.36±0.58c	*Elymus nutans*

The different lowercase letters means significant difference at each metrics (*P*<0.05). Average height of individuals means the average height of all plants individuals in the quadrats. Community height means the height of the tallest plants in the quadrats.

### 2.3 Data analysis

The species importance value (*IV*), which represented the relative importance of species in a community, was calculated using the following equation:

$$IV=\frac{relative\;cover+relative\;height+relative\;density}{3}$$

The Jaccard index (*JI*) means the percentage of a pair species coexist in a quadrat, and it could be used to test interspecific association degree. *JI* was conducted based on the 2 × 2 contingency tables by the plant investigation data (Table 2).

**Table 2:** Illustration of the 2×2 contingency tables.

	Quadrats number of species j appeared	Quadrats number of species j absented
**Quadrats number of species i appeared**	a	b
**Quadrats number of species i absented**	c	-

$$JI=\frac{a}{a+b+c}$$

The *JI* value was classified into 4 grades: none association, 0≦*JI*≦0.25; weak association, 0.25<*JI*≦0.5; middle association, 0.5<*JI*≦0.75; and song association, 0.75<*JI*≦1.0 [29].

Furthermore, the Spearman rank correlation (*r*(*i*, *k*)) was tested to assess the interspecific correlation degree.

$$r(i,k)=1-\frac{6\sum_{j=1}^{N}{\left(x_{ij}-x_{kj}\right)}^{2}}{N^{3}-N}$$

Here, *r(i*, *k)* is the correlation coefficient between species *i* and *k*, *x*_*ij*_ and *x*_*kj*_ are the importance values of species *i* and *k* in quadrat *j*.

Niche theory has been widely used in the study of plant community ecology [30]. Niche breadth and overlap are important indices to further quantify the resource utilization efficiency and competition/coexistence of different populations [31–33]. Shannon-Wiener niche breadth (*B*_*i*_) was calculated following Colwell & Futuyma [34] and Pianka niche overlap (*O*_*ik*_) was calculated using the following equation [35]:

$$B_{i}=-\sum_{j=1}^{r}(P_{ij}\;\ln\;P_{ij})$$

Pij=nijNij

$$O_{ik}=\frac{\sum_{j=1}^{r}P_{ij}P_{kj}}{\sqrt{{\left(\sum_{j=1}^{r}P_{ij}\right)}^{2}{(\sum_{j=1}^{r}P_{kj})}^{2}}}$$

Here, *P*_*ij*_ and *P*_*kj*_ are a proportion of quadrat *j* among the total quadrats occupied by species *i* and *k*; *r* is the total number of quadrats. The n_*ij*_ is the importance values of species *i* in quadrat *j* and *N_ij_* = ∑*n_ij_*.

The soil metric and species data were calculated using MS Excel 2010, and statistical analyses were performed using SPSS Statistics 20.0 (SPSS Inc., Chicago, IL, US). Significant difference between groups were identified taking *P<*0.05 as significant. The soil condition graphs were run with OriginPro 2016 (OriginLab Corporation, Northampton, MA, US). The speed flow distribution characteristics were simulated by Gambit 2.4, Fluent 16.0, and Tecplot 360. The niche overlap matrix diagram was run with the ‘Lattice’ package in R. The Jaccard interspecific association graphs, niche overlap matrix diagrams, and field experimental site pictures were merged by Adobe Photoshop CS6 v6.0.335.0.

## 3. Results

### 3.1. Plant composition and soil conditions in different restoration measures

We recorded 9, 12, and 10 plant species in the sandbag, wicker and CK measures, respectively. The same species importance values varied in the different restoration measures. The *E*. *nutans* occupied the dominant position in all measures, especially in the sandbag measure where the importance value of *E*. *nutans* was up to 52.89 (Table 3).

**Table 3:** Plant composition, importance values (*IV*) and niche breadth (*Bi*) in different restoration measures in August of 2016.

sandbag			wicker			CK		
Species	*IV*	*Bi*	Species	*IV*	*Bi*	Species	*IV*	*Bi*
*En*.	52.89	1.55	*En*.	32.03	1.54	*En*.	39.57	1.55
*Cm*.	17.04	1.54	*Ls*.	19.43	1.48	*Cm*.	19.64	1.42
*Ls*.	10.86	1.50	*Hb*.	12.21	1.50	*Kr*.	17.77	1.46
*Hb*.	8.19	1.41	*Of*.	10.98	1.40	*Ls*.	7.48	1.17
*Of*.	7.78	1.39	*Hl*.	7.61	1.40	*Pb*.	4.38	0.91
*Fo*.	1.43	0.69	*Cm*.	7.48	1.11	*Hb*.	4.20	1.07
*Dh*.	1.27	0.82	*Kr*.	5.52	1.24	*Hl*.	2.28	0.76
*Ms*.	0.31	0.48	*Am*.	1.27	0.58	*Of*.	2.03	0.48
*Od*.	0.23	0.30	*Ps*.	1.24	0.38	*Dh*.	1.82	0.69
			*Ms*.	0.87	0.77	*Ms*.	0.82	0.48
			*Sc*.	0.84	0.48			
			*Ap*.	0.54	0.48			

En., Elymus nutans; Cm., Carex moorcroftii; Kr., Kobresia robusta; Ls., Ligusticum scapiforme; Pb., Potentilla bifurca; Hb., Heteropappus bowerii; Hl., Hypecoum leptocarpum; Of., Oxytropis falcate; Dh., Dracocephalum heterophyllum; Ms., Microula sikkimensis; Am., Artemisia macrocephala; Ps., Polygonum sibiricum; Sc., Salsola collina; Ap., Axyris prostrata; Fo., Festuca ovina; Od., Oxytropis densa.

The soil moisture and nutrient conditions increased greatly in the sandbag and wicker measures than that in the CK (*P<*0.05). Soil moisture in the sandbag measure was 16.67% higher than that in the wicker measure, indicating that the sandbag was more favorable to soil moisture maintains. Analogously, most of the tested nutrients in the sandbag measure were greatly higher than that in the wicker measure. These variances in the different measures indicated that both sandbag and wicker measures could improve the sand dune soil conditions. Moreover, the sandbag measure showed a better improvement on the soil conditions than the wicker measure (Table 4).

**Table 4:** The concentrations of soil moisture and nutrients in different restoration measures in August of 2016 (*P*<0.05).

	Moisture/ %	TN / g kg^-1^	TP/ g kg^-1^	AN/ mg kg^-1^	NN/ mg kg^-1^	AP/ mg kg^-1^	SOC/ g kg^-1^	MBC/ mg kg^-1^	MBN/ mg kg^-1^
**sandbag**	4.11± 0.02a	0.19± 0.02a	0.30± 0.00 a	24.93± 2.04a	7.68± 0.24a	84.05± 0.24a	2.19± 0.10a	65.83± 0.31a	6.23± 0.14a
**wicker**	3.60± 0.02b	0.10± 0.01b	0.33± 0.01a	22.19± 1.24a	5.78± 0.50b	73.37± 0.34b	1.66± 0.09b	52.37± 0.17b	3.53± 0.14b
**CK**	2.81± 0.08c	0.06± 0.00b	0.22± 0.02b	13.39± 0.55b	3.07± 0.74c	43.80± 0.71c	0.95± 0.03c	15.27± 0.60c	0.67± 0.07c

The different lowercase letters means the significant difference at the 0.05 level at each soil metrics (*P*<0.05).

**Fig 2:**
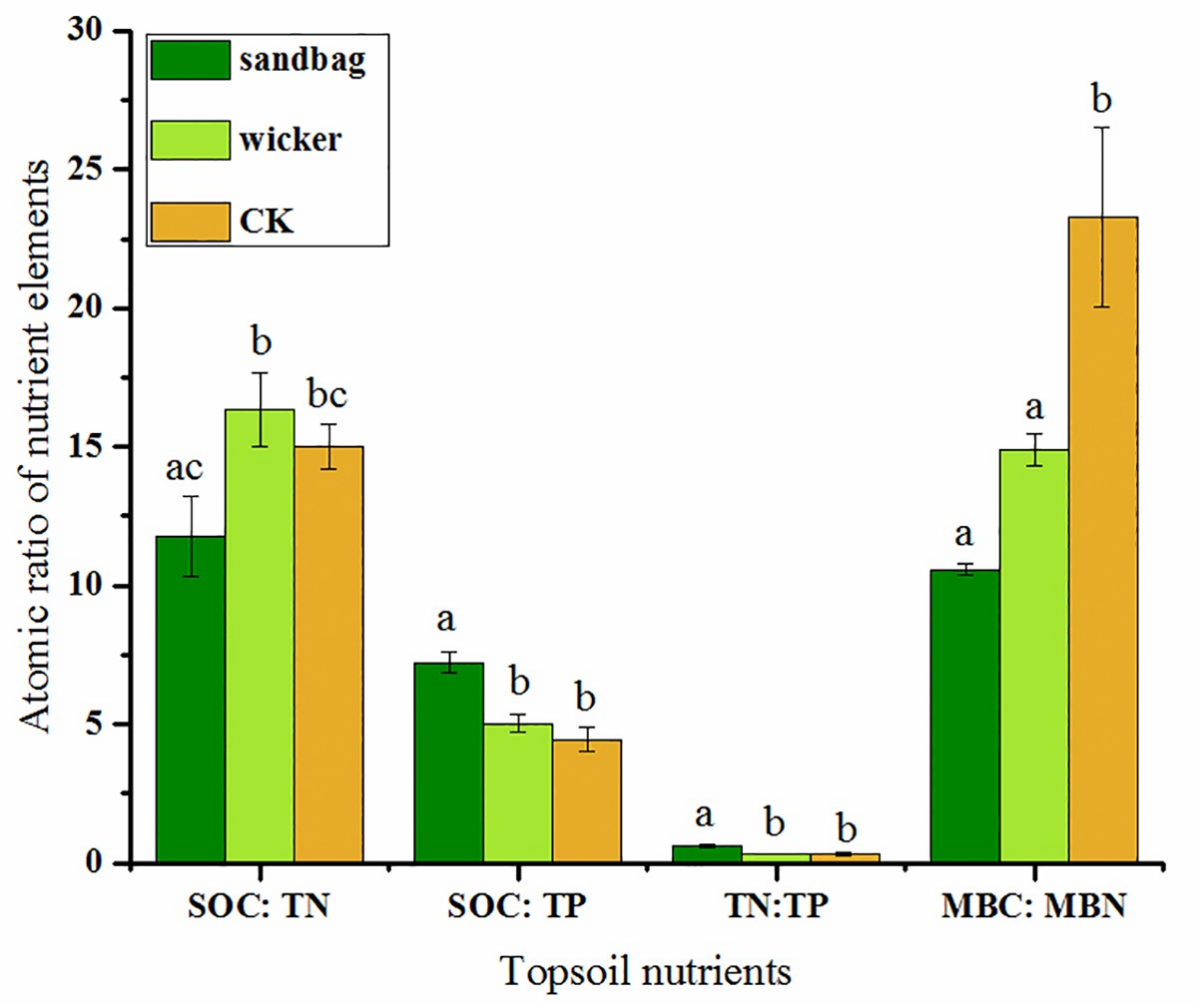
The atomic ratios of SOC, TN, TP, MBC and MBN in different restoration measures in August of 2016. The different lowercase letters means the significant difference at each nutrient pair-wise (*P*<0.05).

The atomic ratios of SOC: TN, SOC: TP, TN: TP and MBC: MBN varied in different restoration measures. The SOC: TN ratio in the sandbag measure was the lowest among the three measures (*P<*0.05). The SOC: TP and TN: TP ratios of the sandbag measure were the highest among the three measures (*P<*0.05). Specifically, the SOC: TP ratio increased by 13.03% and 62.24% and the TN: TP ratios increased by 3.33% and 110.00% in the wicker and sandbag measures compared that in the CK. The MBC: MBN ratios decreased greatly in the wicker and sandbag measures when compared with the CK (*P<*0.05). Though there was insignificant difference of MBC: MBN between the sandbag and wicker measures, it decreased by 28.97% in the sandbag compared that in the wicker measure (Fig 2).

## 3.2. Interspecific relationship in different restoration measures

**Fig 3:**
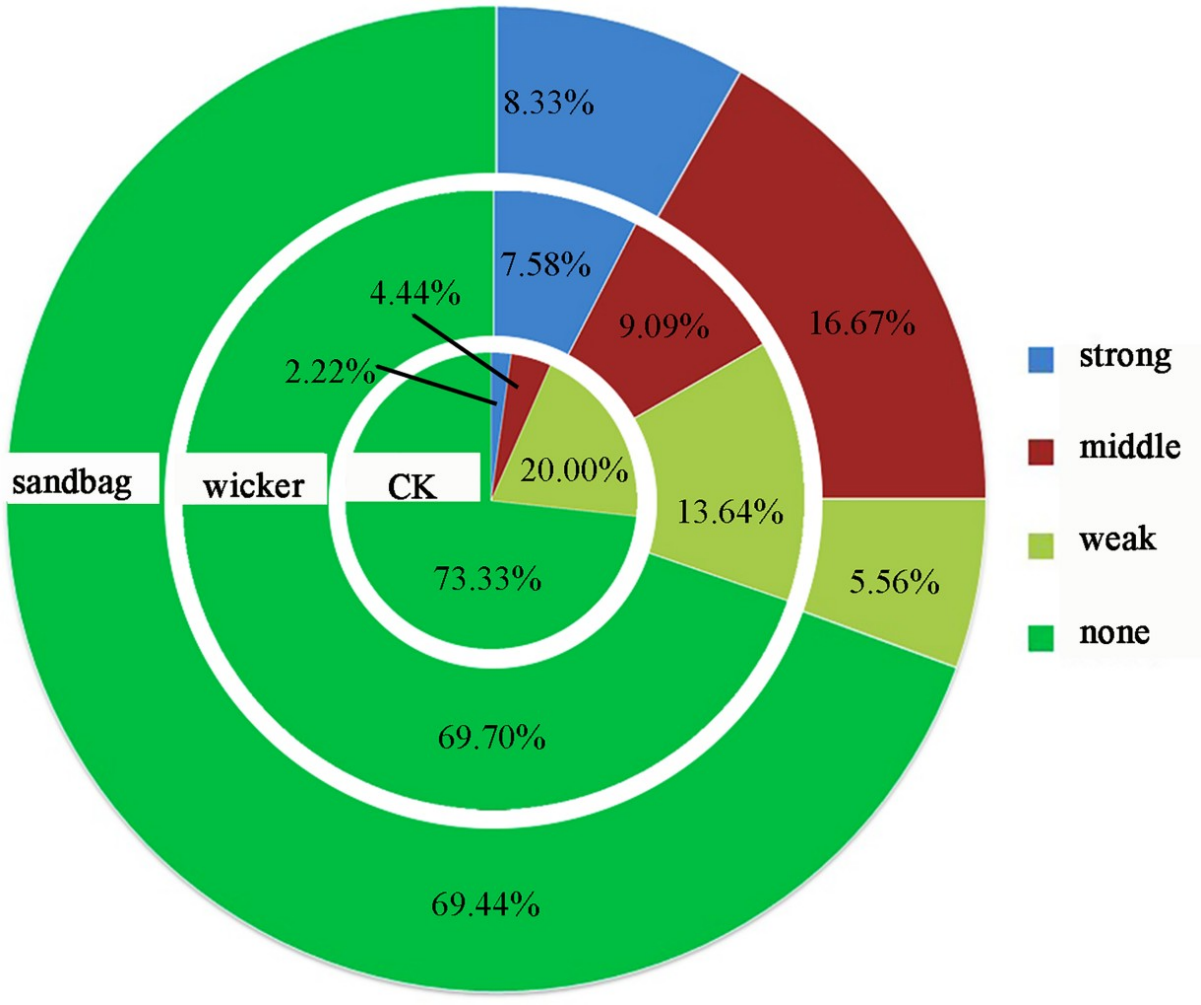
The Jaccard interspecific association (*JI*) ratios in different restoration measures in August of 2016.

The summed ratio of none and weak associations were 75.00%, 83.33% and 93.33% in the sandbag, wicker, and CK measures, respectively. The strong association ratios increased by 241.44% and 275.23% and middle association ratios increased by 104.73% and 275.45% in the wicker and sandbag measures when compared with the CK (Fig 3). The plant community was the simplest structured in the CK. The sandbag measure caused a closer associated community than the wicker measure. It indicated that the community development status was better in the sand dunes that amended by the sand barriers.

The negative Spearman rank correlation indices ratios were 75.00%, 63.63% and 68.89% in the sandbag, wicker and CK measures, respectively. *E*. *nutans* was negatively correlated with most of plants, accounting for 87.50%, 72.73% and 66.67% of all species pairs in the sandbag, wicker and CK measures, respectively. In addition, the same species pair interspecific correlation changed in the different restoration measures. For example, *E*. *nutans*-*C*. *moorcroftii* changed from greatly negative correlation to positive correlation (i.e., -0.47 in the CK (*P*<0.01), -0.18 in the wicker, and 0.10 in the sandbag); and the negative correlation degree of *E*. *nutans*-*H*. *bowerii* was enhanced by the sand barriers measures (-0.09 in the CK, -0.34 (*P*<0.05) in the wicker, and -0.47 (*P*<0.01) in the sandbag) (Tables 5–7).

**Table 5:** Spearman rank correlations of species pairs in the sandbag measure in August of 2016.

	*En*	*Cm*	*Ls*	*Hb*	*Of*	*Fo*	*Dh*	*Ms*
*Cm*	0.096**							
*Ls*	-0.217	-0.149						
*Hb*	-0.466**	-0.050	-0.266					
*Of*	-0.006	-0.489**	-0.318	-0.189				
*Fo*	-0.055	0.113	-0.528**	0.148	-0.081			
*Dh*	-0.339*	-0.363*	0.629**	-0.071	-0.246	-0.195		
*Ms*	-0.470**	-0.254	-0.442**	0.393*	0.395*	0.508**	-0.147	
*Od*	-0.108	0.203	-0.265	0.190	-0.084	-0.097	-0.118	-0.073

*, Correlation is significant at the 0.05 level (*P*<0.05)

**, Correlation is extremely significant at the 0.01 level (*P*<0.01).

**Table 6:** Spearman rank correlation of species pairs in the wicker measure in August of 2016.

	*En*	*Ls*	*Hb*	*Of*	*Hl*	*Cm*	*Kr*	*Am*	*Ps*	*Ms*	*Sc*
*Ls*	-0.128										
*Hb*	-0.336*	-0.345*									
*Of*	-0.150	0.225	-0.237								
*Hl*	-0.269	-0.196	0.005	-0.275							
*Cm*	-0.179	-0.689**	0.526**	-0.214	0.053						
*Kr*	0.337*	0.395*	-0.488**	-0.162	-0.047	-0.747**					
*Am*	-0.416*	-0.145	0.195	-0.151	0.304	-0.054	-0.147				
*Ps*	-0.359*	-0.344*	0.180	-0.075	0.242	0.175	-0.279	0.585**			
*Ms*	-0.511**	0.022	0.143	0.218	0.153	0.099	-0.119	-0.157	-0.134		
*Sc*	0.202	-0.478**	0.339*	-0.394*	-0.394*	0.533**	-0.279	-0.106	-0.091	-0.134	
*Ap*	0.363*	-0.172	-0.479**	0.196	0.060	-0.243	0.348*	-0.106	-0.091	-0.134	-0.091

*, Correlation is significant at the 0.05 level (*P*<0.05)

**, Correlation is extremely significant at the 0.01 level (*P*<0.01).

**Table 7:** Spearman rank correlation of species pairs in the CK in August of 2016.

	*En*	*Cm*	*Kr*	*Ls*	*Pb*	*Hb*	*Hl*	*Of*	*Dh*
*Cm*	-0.465**								
*Kr*	0.102	-0.614**							
*Ls*	-0.314	-0.277	0.068						
*Pb*	-0.019	-0.181	0.405*	-0.241					
*Hb*	-0.085	0.405*	-0.487**	-0.380*	-0.392*				
*Hl*	0.118	0.101	-0.455**	-0.359*	-0.253	0.701**			
*Of*	0.278	0.312	-0.436**	-0.243	-0.172	-0.207	-0.134		
*Dh*	-0.207	-0.326	0.246	0.596**	-0.228	-0.275	-0.178	-0.121	
*Ms*	-0.376*	0.082	0.027	0.224	-0.172	-0.207	-0.134	-0.091	-0.121

*, Correlation is significant at the 0.05 level (*P*<0.05)

**, Correlation is extremely significant at the 0.01 level (*P*<0.01).

## 3.3. Niche breadth and overlap in different restoration measures

Population niche breadth and niche overlap analyses could effectively assess resources utilization and interspecific competition. The niche breadth ranged from 0.29 to 1.55 in the sandbag measure, 0.38 to 1.54 in the wicker measure, and 0.48 to 1.55 in the CK measure. *E*. *nutans* had the widest niche breadth in the all measures. And the drought resistance plants, such as *H*. *bowerii*, and *C*. *moorcroftii*, also occupied a wide niche breadth in the community. Furthermore, sand barriers provided a possibility for some other species dispersing and settling down. Such as the *P*. *sibiricum*, *S*. *collina* and *F*. *ovina* were absent from the CK but presented in the wicker or sandbag measures (Table 3).

**Fig 4:**
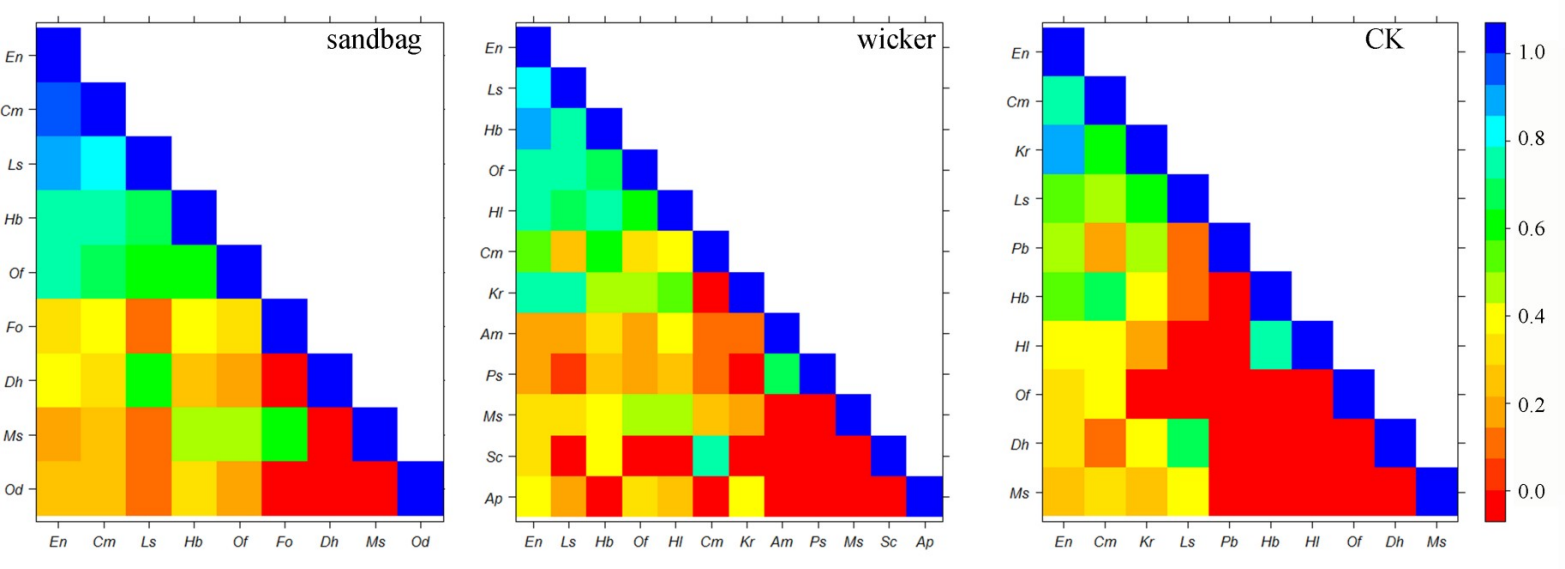
Niche overlap of all plant pairs in different restoration measures in August of 2016.

The average population niche overlap indices in the sandbag, wicker, and CK measures were 0.39, 0.32 and 0.26, respectively. Furthermore, the species-pair ratio that niche overlap was higher than 0.50 accounting for 33.33%, 25.76% and 20.00% in the sandbag, wicker and CK measures, respectively. The increased niche overlap indicated that the interspecific competition was stronger under the sand barriers amendment. Moreover, a stronger improvement effect of sandbag measure than wicker measure on plant community could be inferred in this study (Fig 4).

## 4. Discussion

It is proved to be practical that incorporation of indigenous grass seeding and sandbag or wicker sand barriers measures on the alpine sand dune restoration. Different restoration measures led to obvious different soil conditions. The soil was extremely droughty in the sand dune, especially in the land that without sand barrier [36]. However, the different porosity between sandbag and wicker may lead to different near-surface wind velocities [37, 38] (S1 Fig). The lower wind velocities would protect the soil water from being blown away. Thus, the extremely droughty was improved greatly after the sand barriers were established.

Except for the soil moisture condition was improved, the nutrients also got improved in the sand barrier measures. Though the soil nutrients in the all measures were more barren than that in the Zoige wetland (e.g., the TN ranged from 4.9 to 12.0 g kg^-1^), they were greatly improved by the wicker or sandbag measures [39]. The greatly increased microbial mass indicated higher microbe richness in the amended sand dune soil. Hence, it accelerated the litter decomposition in the soil and fed back nutrients increasing [40]. For example, the nitrogen content increasing in the wicker and sandbag measures, and the SOC: TN ratio in the sandbag measure decreased to close to the level in the Zoige meadow (SOC: TN = 11.8) [41]. Nonetheless, the SOC: TP and TN: TP ratios were still lower than that in the meadow, and this result may indicate a phosphorus inhibition on the sand dune communities [42]. These changes suggested that the nutrients (e.g., nitrogen) inhibition degree was reduced in the wicker and sandbag measures. Hence, it could be inferred that the incorporation of indigenous grass and sand barriers contributed to soil nutrients improvements. And the improvements of soil would accelerate the vegetation restoration [43, 44].

The vegetation restoration process is accompanied with the plant interspecific relationship and niche changes. Plants interspecific relationship or niche play a critical role in stabilizing community [30, 45, 46]. And it is important for revealing how species interact with each other and adapt with the environment. Hence, it has important implications for optimal restoration measures in degraded ecosystems [45]. The interspecific association degree was enhanced in the wicker or sandbag measures. The closer interspecific correlation and higher niche overlap reflected a stronger competitive relationship under the sand barriers treatments [47]. The population space occupancy and correlation degree was the lowest in the CK while the highest in the sandbag measure. Hence, the sandbag measure may lead to the best plant community development in sand dune restoration [37]. Though some previous studies stated that interspecific competition/association degree reduced gradually with the plant community development [48, 49], there was an increasing tendency of species competition and association degree with the increase of the vegetation cover in this study. The reason may be that the original plant community was extremely limited structure and minimal resource acquisition ability, thus the independence between vascular plants was strong in such barren sand dune [50]. Thus, the interspecific association among the plants got closer along with the vegetation restoration process.

Plant population niche and structure indicate the orientation of plant community development well [51], and reveal community assembly mechanism quantitatively [49, 52]. Plant species survived under different restoration measures, and the population niche and interspecific relationships changed along the changed environment [49, 53–55]. Resource variations resulted in different ecological strategies among different plant populations, and these plants existed mutual effect with each other [50, 56, 57]. The *E*. *nutans* importance value increased in the sand barrier measures, especially in the sandbag measure. And it occupied the widest niche which allowed it to compete for the soil and light resource or coexist with others. For example, the relationship of *E*. *nutans*-*C*. *moorcroftii* changed from greatly negative to positive correlation; the negative correlation degree of *E*. *nutans*-*H*. *bowerii* was enhanced in the wicker and sandbag measures. Thus, it suggested that the sown grass could regulate the community structure and promote the vegetation restoration. Meanwhile, we also suggested that it is important to restore sand dunes by developing a community with different ecological strategies preferentially.

## 5. Conclusions

The alpine sand dunes restoration by incorporating the sand barriers and indigenous grass could improve the soil conditions and community structure. The sandbag and wicker measures would gain a better restoration effect than that only seeding. Moreover, sandbag measure allowed for a more prominent restoration effect on the harsh soil conditions and plant community than wicker measure. And the sandbag measure could utilize the sand soil in the restoration area. Hence, we suggested that the sandbag measure should be popularized in the alpine sand dune restoration. Also, we suggested that the interspecific relationships, niche characteristics and soil conditions could be used to assess the effect of sand dune restoration.

## Supporting information

S1 Fig The speed flow distribution characteristics of different restoration measure. (DOCX)

S1 Dataset Soil conditions and vegetation conditions. (XLSX)
