# Supplementary figures and images for: Soil and vegetation conditions changes following the different sand dune restoration measures on the Zoige Plateau
Source: PLoS One. 2019 Sep 20;14(9):e0216975. doi: 10.1371/journal.pone.0216975 (PMC6754152; doi:10.1371/journal.pone.0216975)

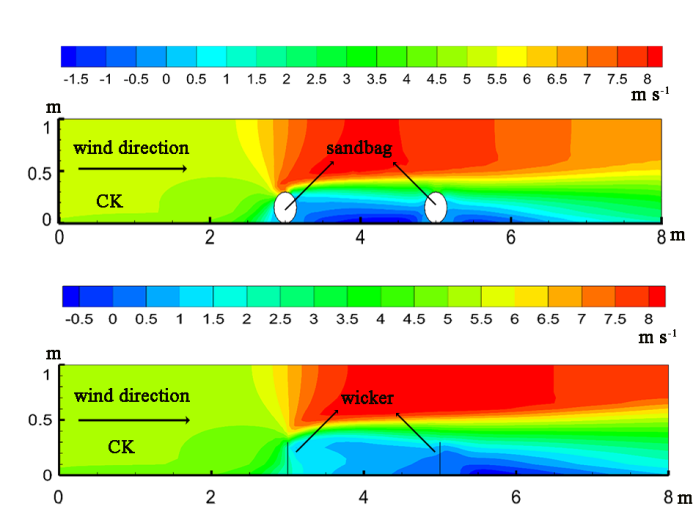


**S1 Fig. The speed flow distribution characteristics of different restoration measure.**

Supplement: S1 Fig — (DOCX) [file pone.0216975.s001.docx]
